# Ethylicin Prevents Potato Late Blight by Disrupting Protein Biosynthesis of *Phytophthora infestans*

**DOI:** 10.3390/pathogens9040299

**Published:** 2020-04-19

**Authors:** Shumin Zhang, Meiquan Zhang, A. Rehman Khalid, Linxuan Li, Yang Chen, Pan Dong, Hanyan Wang, Maozhi Ren

**Affiliations:** 1 School of Life Sciences, Chongqing University, Chongqing 401331, China; zhangshumin@nsmc.edu.cn (S.Z.); zhangmeiquan@cqu.edu.cn (M.Z.); dongpan@cqu.edu.cn (P.D.); 2 School of Basic Medical Sciences, North Sichuan Medical College, Nanchong 637000, China; wanghanyan@nsmc.edu.cn; 3 Department of Plant Pathology, University of Poonch Rawalakot AJK, Rawalakot AJK 12350, Pakistan; arkhalid@cqu.edu.cn; 4 Institute of Urban Agriculture, Chinese Academy of Agricultural Sciences, Chengdu 610000, China; lilinxuan@caas.cn; 5 Research Center for Atmospheric Environment, Chongqing Institute of Green and Intelligent Technology, Chinese Academy of Sciences, Chongqing 400714, China; chenyang@cigit.ac.cn

**Keywords:** *Phytophthora infestans*, ethylicin, potato late blight, proteomics, ribosome, metabonomics, protein biosynthesis

## Abstract

*Phytophthora infestans*, the causal agent of potato late blight, triggered the devastating Great Irish Famine that lasted from 1845 to 1852. Today, it is still the greatest threat to the potato yield. Ethylicin is a broad-spectrum biomimetic-fungicide. However, its application in the control of *Phytophthora infestans* is still unknown. In this study, we investigated the effects of ethylicin on *Phytophthora infestans*. We found that ethylicin inhibited the mycelial growth, sporulation capacity, spore germination and virulence of *Phytophthora infestans*. Furthermore, the integrated analysis of proteomics and metabolomics indicates that ethylicin may inhibit peptide or protein biosynthesis by suppressing both the ribosomal function and amino acid metabolism, causing an inhibitory effect on *Phytophthora infestans*. These observations indicate that ethylicin may be an anti-oomycete agent that can be used to control *Phytophthora infestans*.

## 1. Introduction

*Phytophthora infestans* (*P. infestans*) is a notorious oomycete pathogen, which caused the Great Irish Famine in the mid-19th century [1]. Today, it remains the most serious threat to potato production, leading to huge economic losses worldwide [2]. Over the past decades, the use of chemical oomyceticides has been the main method for controlling *P. infestans*. However, environmental protection and human health and food safety are challenged by the overuse of chemical oomyceticides, leading to the promotion of the development of bio-oomyceticides which are less toxic to the environment and humans. For example, some natural productions such as melatonin and the metabolites of *Trichoderma* have the potential to be developed into bio-oomyceticides by virtue of their significantly inhibitory effects on *P. infestans* [3,4]. The main mechanisms of chemical oomyceticides are focused on destroying the respiratory chain, decreasing the generation of ATP, inhibiting RNA polymerase and suppressing the metabolism of *P. infestans* [5,6,7]. However, compared with chemical oomyceticides, the detailed mechanisms of bio-oomyceticides have not been further explored. Taking melatonin as an example, previous studies that only used transcriptome speculated that melatonin may target the metabolism to suppress *P. infestans*, but the detailed mechanisms were not thoroughly explored. Thus, aside from developing novel bio-oomyceticides, it is also necessary to further explore the underlying mechanisms of bio-oomyceticides in order to promote their application.

Ethylicin (S-Ethyl ethanethiosulfonate; CAS number 682-91-7) is an allicin analogue. China first discovered its broad-spectrum bactericidal efficacy, then developed it into a biomimetic fungicide [8]. It is able to combat various fungi, including *Rhizoctonia solani* Kühn, *Verticillium dahlia* Kleb., *Pyricularia oryae* Cav., *Fusarium graminearum* Sehw., etc. Its anti-fungal mechanism is well-known; the molecular structure of ethylicin contains -S-S (=O)_2_, which disrupts -SH-based molecules in pathogen cells, leading to the inhibition of metabolism in plant pathogens [8]. Additionally, ethylicin can be used as a plant growth regulator for stimulating seed germination, increasing yields, improving quality, etc. [8]. Thus, ethylicin is a biomimetic-fungicide which causes less harm to plant growth. However, it is unknown whether ethylicin can be effective against *P. infestans*, which metabolic process it mainly inhibits and whether it targets additional pathways.

In this study, the effects of ethylicin on *P. infestans* were investigated and the underlying mechanisms analyzed by proteomics and metabonomics. The results show that ethylicin inhibits the mycelial growth, sporulation capacity, spore germination and virulence of *P. infestans*. The integrated analysis of proteomics and metabolomics suggests that ethylicin exhibits an inhibitory effect on peptide or protein biosynthesis via the suppression of ribosomal function and amino acid metabolism. These findings are useful for providing a set of observations on the inhibitory effects of ethylicin on *P. infestans*.

## 2. Materials and Methods

### 2.1. P. infestans Strains, Media and Culture Conditions

*P. infestans* strains T30-4 and 88069 were provided by Dr. Suomeng Dong of Nanjing Agriculture University, China. The T30-4 and 88069 strains were maintained on a Rye A agar medium at 18 °C in the dark, in accordance with a previous study [9].

### 2.2. Effects of Ethylicin on the Mycelial Growth, Sporulation Capacity, Spore Germination and Virulence of P. infestans

A number of 6-mm-diameter T30-4 or 88069 mycelial disks were maintained on Rye A agar plates and supplemented with the following concentrations of ethylicin (80% purity, soluble in water; Bissell agricultural technology company, Henan, China): 0, 32.5, 65 and 130 μM for T30-4 and 88069 strains. Water was used as the control. When the diameters of the control colonies (T30-4 or 88069 strains) reached 6 cm, the diameters of the ethylicin-treated colonies (T30-4 or 88069 strain) were measured and the inhibition rates calculated. Three biological repeats were performed for each experiment.

Sporangia of T30-4 or 88069 were washed from the plates of ethylicin-treated and water-treated colonies and then counted. Next, sporulation capacity was estimated using the following calculation formula: sporulation capacity (sporangia per unit area) = total sporangia number of drug-treated colony/area of drug-treated colony. Three biological repeats were performed for each experiment.

Sporangia of T30-4 or 88069 were first collected by washing off the plates with water, and the concentration of spore suspension was adjusted to 40 spores/μL. Next, the spore suspensions of T30-4 or 88069 were supplemented with the following ethylicin concentrations (0, 6.5, 19.5, 32.5 and 65 μM for T30-4 and 88069 strains). Water was used as the control. The spore germination of the two strains (T30-4 and 88069) was then counted after being cultured at 18 °C for 12 h. Both germinated sporangia and zoospores were counted. Three biological repeats were performed for each experiment.

For the virulence assay on tubers, the preparation procedures of spores have been described in the spore germination procedures. The concentration of the spore suspension was 40 spores/μL. The spore suspensions of T30-4 or 88069 strain were pretreated with the following ethylicin concentrations: 0, 6.5, 32.5, 65 and 195 μM for T30-4 and 88069. Then, the whole potato tuber (cultivar: Favorita) was cut into regular slices (4 × 6 cm). Next, 20 µL of ethylicin-treated or control-treated spore suspension (T30-4 or 88069) was inoculated on the tuber slices for 4 days at 18 °C with a 12-h/12-h light/dark cycle. After 4 days, the size of the lesions on the potato tubers was measured. Three biological repeats were performed for each experiment.

For the virulence assay on potato plants, potato plants (cultivar: Favorita) were grown under greenhouse; culture conditions: 18 ℃, 16-h/8-h light/dark cycle, 60–90 μE·m^−2^·s^−1^ light intensity and 90% humidity. The spore preparation procedures of T30-4 or 88069 have been described in the spore germination procedures (spore suspension concentration: 40 spores/μL). The potato plants were then sprayed with spores of T30-4 or 88069. After 12 h, the inoculated potato plants were sprayed with different concentrations of ethylicin (0, 19.5, 130 and 195 μM for T30-4; 0, 19.5, 65 and 195 μM for 88069). Next, the inoculated plants were cultured for 10 days. After 10 days, the disease index and prevention effects were measured. Three biological repeats were performed for each experiment.

### 2.3. Proteomics Assay and Dataset Analysis

The T30-4 was cultured in a liquid Rye A agar medium for 14 days. The mycelia of T30-4 were then treated by water and 65 μM of ethylicin (IC50) for 24 h at 18 °C in the dark. After collecting the mycelia, they were frozen in liquid nitrogen for protein extraction. Next, a proteomics assay was performed by PTM-BIO of Hangzhou, China. A lysis buffer was used to extract the total proteins from the mycelia. Then, proteins were digested by trypsin into peptides. After digestion and reconstitution, the protein samples were turned into fractions using high pH reverse-phase HPLC. Next, the peptide samples were dissolved, loaded onto reverse-phase analytical columns and subjected to an NSI source. The purpose of NSI was to charge the peptide segment before it can be identified by the mass spectrometer. Three biological repeats were performed for each experiment. UniProt was the reference genome database. Website: https://www.uniprot.org/. Proteins with more than a 1.2-fold increase (E/DMSO Ratio > 1.200) or decrease (E/DMSO Ratio < 0.833) and with a *p*-value < 0.05 were recognized as differentially expressed proteins (DEPs), and GO terms and KEGG pathways with a *p*-value < 0.05 were recognized as significant. *p*-Values were calculated by two-tailed Student’s *t*-test analysis, which was performed using the IBM SPSS statistics software. The main equipment included HPLC (Thermo Scientific, Waltham, MA, USA), 300Extend-C18 (Agilent, Santa Clara, CA, USA), Q ExactiveTM Plus (Thermo Scientific), etc. The software used for statistical computations included MaxQuant for mass spectrum data analysis, MoMo for motif analysis, Interproscan for GO term and domain analysis, KAAS and KEGG Mapper for KEGG analysis, Wolfpsort and CELLO for subcellular localization. The versions and websites of these software: MaxQuant: v.1.5.2.8 (http://www.maxquant.org/); MoMO: V5.0.2 (http://meme-suite.org/tools/momo); Interproscan: v.5.14-53.0 (http://www.ebi.ac.uk/interpro/); KAAS: v.2.0 (http://www.genome.jp/kaas-bin/kaas_main); KEGG Mapper: V2.5 (http://www.kegg.jp/kegg/mapper.html); Wolfpsort: v.0.2 (http://www.genscript.com/psort/wolf_psort.html); CELLO: v.2.5 (http://cello.life.nctu.edu.tw/).

### 2.4. Metabonomics Assay and Dataset Analysis

The sample preparation procedures have been described in the proteomics assay procedures. A metabonomics assay was performed by Metabo-Profile of Shanghai, China. The XploreMET platform was used to perform untargeted metabolomic profiling. The samples were treated according to the following steps: centrifugation, extraction, evaporation, lyophilization, derivatization and injection. The following instrumentation was used for separation, measurement and analysis: time-of-flight mass spectrometry system (Pegasus HT, Leco Corp., St. Joseph, MO, USA) coupled with gas chromatography (Agilent 7890B, Santa Clara, CA, USA) and a Gerstel MPS2 MultiPurpose Sampler (Gerstel, Mülheim, Germany). Four kinds of quality control sampling were performed on the comprehensive metabolomics platform, namely test mixtures, retention indices, internal standards and pooled biological QC samples. For metabolite annotation, the retention indices and mass spectral data were compared with the data generated from the reference standards in the JiaLib metabolite database (Metabo-Profile, Shanghai, China) using Xplore MET. The data analysis included the following six parts: raw mass spectral data processing, data preprocessing, ratio generation, statistics, multivariate statistical analyses and univariate statistical analyses. VIP (variable importance in projection) and Corr.Coeffs (correlation coefficient) were obtained based on the OPLS-DA model. The *p*-values were calculated by Spearman correlation analysis, which was performed using the IBM SPSS statistics software. Metabolites with VIP of ≥1 and *p*-value <0.05 were regarded as statistically significant (differentially expressed metabolites: DEMs). Corr.Coeffs represents variable reliability; if the value is closer to 1 or −1, then the reliability is higher. The software used for statistical computations included Xplore MET for mass spectrum data analysis, R packages ropls for PCA and PLS-DA and OPLS-DA, Metaboanalyst for KEGG pathway analysis. The versions, websites or providers of these software: 

Xplore MET: V2.0 (Metabo-Profile, Shanghai, China); R packages ropls: V1.18.8 (http://www.bioconductor.org/packages/release/bioc/html/ropls.html); Metaboanalyst: V4.0 (https://www.metaboanalyst.ca/). 

### 2.5. Protein Content Test

The T30-4 and 88069 strains were cultured in a liquid Rye A agar medium for 14 days. The mycelia of T30-4 or 88069 were then treated with different concentrations of ethylicin (0, 32.5, 65, 130 and 195 μM for T30-4; 0, 195, 325, 455 and 650 μM for 88069) for 24 h at 18 °C in the dark. Then, the mycelia were collected, dried in an oven, weighed and frozen in liquid nitrogen to extract the total protein. Next, protein quantification was performed using a BCA test (Beyotime biotechnology of Shanghai, China). Protein content (% DW) = [total protein weight (mg)/mycelia dryweight (mg)] × 100%. 

## 3. Results

### 3.1. Ethylicin Significantly Affects P. infestans Mycelial Growth, Sporulation Capacity, Spore Germination and Virulence

**Figure 1:**
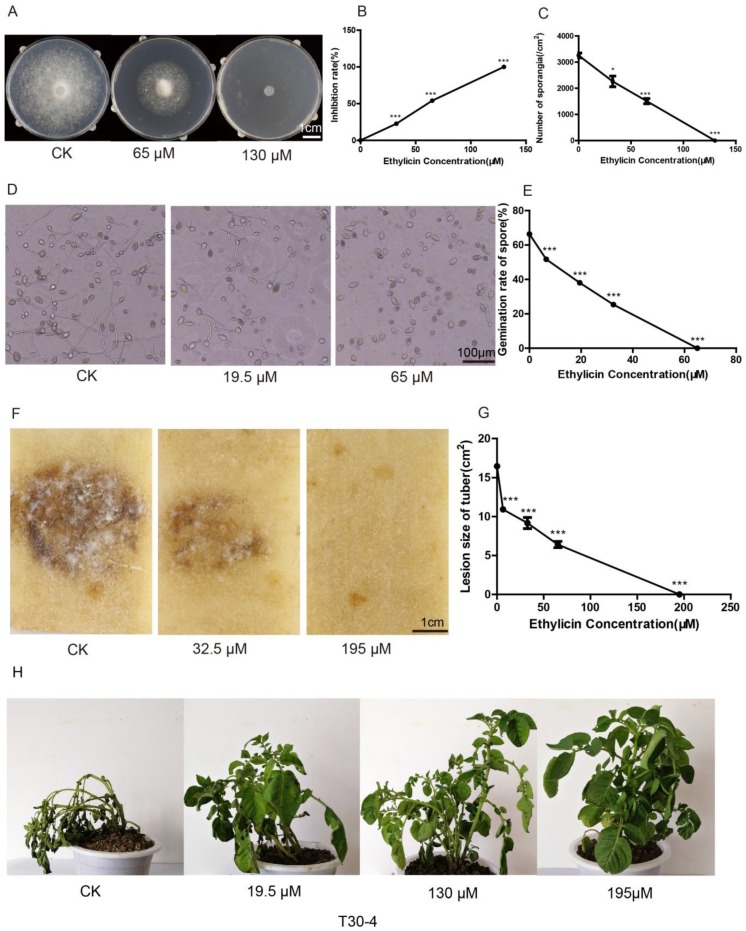
Ethylicin significantly inhibits *P. infestans* (T30-4). (**A**) Mycelial growth of T30-4 treated with different concentrations of ethylicin for 14 days. A quantity of 65 μM of ethylicin was the IC50 value, while 130 μM completely inhibited mycelial growth. (**B**) Inhibition rates of ethylicin against mycelial growth for 14 days. (**C**) Statistics of sporulation capacity under treatment by different concentrations of ethylicin. (**D**) Spore germination phenotype of T30-4 under treatment by different concentrations of ethylicin. A quantity of 19.5 μM of ethylicin showed a 50% inhibitory effect on spore germination, while 65 μM nearly completely inhibited spore germination. (**E**) Spore germination rates of T30-4 under treatment by different concentrations of ethylicin. (**F**) Tubers of potato cultivar Favorita were inoculated with spore solution (T30-4). Spore solution was pretreated with different concentrations of ethylicin and then inoculated on potato tubers. (**G**) Lesion size of tubers under treatment by different concentrations of ethylicin for 4 days. 32.5 μM of ethylicin was the IC50 value; 195 μM nearly completely alleviated disease symptoms. (**H**) Disease symptoms of potato plants inoculated with T30-4. Potato plants were first sprayed with spores of T30-4, then sprayed with different concentrations of ethylicin. Disease symptoms were documented after 10 days. * *p* < 0.05, ** *p* < 0.01, *** *p* < 0.001. *p*-Values were calculated by two-tailed Student’s *t*-test analysis. Each value represents the mean ± SD of three independent experiments.

To determine whether ethylicin would attenuate potato late blight, we investigated the inhibitory effects on *P. infestans* (T30-4 and 88069 strains) following ethylicin treatment. The results show that ethylicin exhibits an inhibitory effect on mycelial growth, sporulation capacity, spore germination and virulence with dose-dependent effects on the strains of T30-4 and 88069 (Figure 1 and Supplementary Figure S1). The mycelial growth, sporulation capacity, spore germination and virulence on tubers were 50% inhibited (IC50) by ethylicin treatment at concentrations of around 65, 65, 19.5 and 32.5 μM for T30-4 (Figure 1A–G) and 65, 65, 19.5 and 32.5 μM for 88069 respectively (Supplementary Figure S1A–G). 

Next, the anti-*P. infestans* effects of ethylicin on the overall potato plant were tested. The potato plants were first inoculated with T30-4 or 88069, then sprayed with different concentrations of ethylicin. After 10 days, potato plants inoculated with T30-4 but without ethylicin treatment exhibited obvious disease symptoms, whereas the inoculated potato plants that were treated with increasing concentrations of ethylicin showed reduced disease lesions (Figure 1H); this was confirmed by the decreased disease index and increased prevention effects (Supplementary Table S1). Similar performance was observed in the potato plants inoculated with 88069 and treated with ethylicin (Supplementary Figure S1H and Supplementary Table S1). However, the control effects of plants first sprayed with ethylicin for 24 h and then inoculated with spores for 12 h were not as good as those achieved by the above-mentioned method (data not shown). Thus, these results indicate that ethylicin inhibits potato late blight. 

### 3.2. Analysis of Inhibitory Mechanisms of Ethylicin on P. infestans 

It is well known that ethylicin fights plant pathogens by targeting the metabolism [8]. However, such questions as which kind of metabolic process is mainly targeted by ethylicin and whether ethylicin may impact additional pathways in plant pathogens remain unexplored. To further dissect the underlying mechanisms of ethylicin against *P. infestans*, proteomic and metabolomic analysis was performed on ethylicin-treated *P. infestans*.

#### 3.2.1. Ethylicin May Target Amino Acid Metabolism to Inhibit Protein Biosynthesis in *P. infestans*

**Figure 2:**
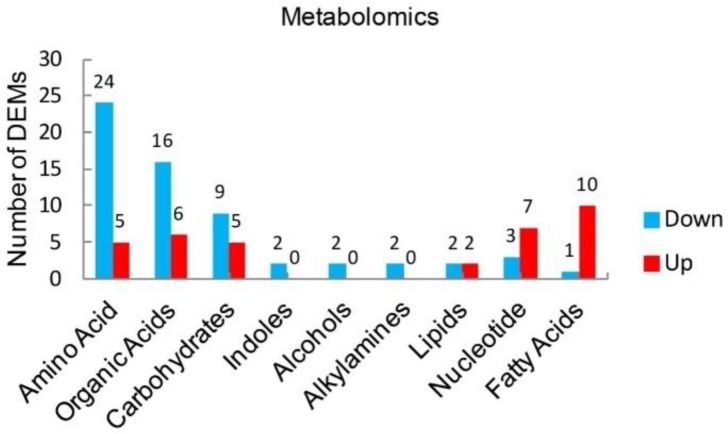
Nine classes of metabolites showed differential expression in *P. infestans* in response to ethylicin. The most abundant DEMs were amino acids, followed by carbohydrates and organic acids. The number of total DEMs in amino acids was 1.3-fold, 2.1-fold, 14.5-fold, 14.5-fold, 14.5-fold, 7.25-fold, 2.9-fold and 2.6-fold that of organic acids, carbohydrates, indoles, alcohols, alkylamines, lipids, nucleotides and fatty acids respectively. The number on top of the histograms means the number of up or down-regulated DEMs in nine classes of metabolites. Up-regulated DEMs: FC > 1, down-regulated DEMs: FC < 1.

In order to explore which metabolic process is mainly targeted by ethylicin, various differentially expressed metabolites (DEMs) in metabolomics were analyzed after ethylicin treatment. The metabolomic data shows that three classes of metabolites were not significantly changed after ethylicin treatment: vitamin, phosphates and aldehydes (Supplementary Table S2A). However, 12 classes of DEMs showed differential expression after ethylicin treatment: amino acids (29 kinds), organic acids (22 kinds), carbohydrates (14 kinds), nucleotides (10 kinds), alcohols (2 kinds), alkylamines (2 kinds), fatty acids (11 kinds), indoles (2 kinds) and lipids (4 kinds) (Figure 2, Supplementary Table S2B). Among these 12 classes of DEMs, the most abundant were amino acids, suggesting that ethylicin mainly influences the amino acid metabolism of *P. infestans* (Figure 2, Supplementary Table S2B). Further analyses indicated that up-regulated DEMs were predominant in fatty acids (up: 10, down: 1) and nucleotides (up: 7, down: 3) (Figure 2, Supplementary Table S2B and Supplementary Table S3), indicating that not all metabolic processes are inhibited by ethylicin. The down-regulated DEMs were predominant in amino acids (up: 5, down: 24), carbohydrates (up: 5, down: 9) and organic acids (up: 6, down: 16) (Figure 2, Table 1, Supplementary Table S2B and Supplementary Table S3). These results show that ethylicin suppresses the above-mentioned metabolic processes, especially amino acid metabolism. Because peptides and proteins are composed of amino acids, the decreased amino acids may inhibit the biosynthesis of peptides or proteins. The main function of carbohydrates is to provide energy, so the decreased carbohydrates may result in a lack of the energy necessary for peptide or protein biosynthesis. All of these factors may contribute to the reduction of peptides and proteins, the executors of life activities, in *P. infestans*.

Collectively, ethylicin may inhibit the metabolic processes of amino acids and carbohydrates, especially amino acid metabolism, resulting in decreased amino acids and energy, and this may cause the inhibition of peptide or protein synthesis in *P. infestans*. 

#### 3.2.2. Ethylicin Inhibits Accumulation of Ribosomal Components

Aside from the metabolism, additional pathways targeted by ethylicin were analyzed by proteomics. In the proteomic assay, 289 DEPs were identified in the ethylicin-treated samples in contrast to the control (Supplementary Table S4). Among them, 113 were significantly up-regulated and 176 down-regulated (Supplementary Table S4). Overall, this indicates that ethylicin inhibits protein expression in *P. infestans*.

To dissect the detailed mechanisms of ethylicin against *P. infestans*, we analyzed the GO terms and KEGG pathways. The total DEPs were divided into three kinds of GO term at level 1 (biological process, cellular component and molecular function), including 26 kinds of child GO terms with *p*-values <0.05 (Table 2, Supplementary Table S5A–D). Among the 26 child GO terms, 10, 8 and 8 child GO terms belonged to biological processes, cellular processes and molecular functions respectively. Notably, ribosome and the structural constituents of ribosome showed the most significant changes of function among cellular processes and molecular functions respectively (sorted by their *p*-values in descending order; the same below; Table 2, Supplementary Table S5A). Furthermore, the down-regulated proteins were predominant in ribosome and the structural constituents of ribosome (Table 2, Supplementary Table S5B,C). In particular, the protein expressions of key DEPs related to ribosome, such as ribosomal protein S9, ribosomal protein L24, ribosomal protein L32, ribosomal protein L27 and ribosomal protein S18, were suppressed by ethylicin (Supplementary Table S5B,C). The knockout of these proteins can damage the functions of ribosome in various species [10,11,12,13,14]. Thus, these results suggest that the ribosomal functions of *P. infestans* may be inhibited by ethylicin. Further analysis of the KEGG pathways showed that ribosome was the most significant change of pathway under ethylicin treatment (Table 3, Supplementary Table S6A), and all of the DEPs related to ribosome were down-regulated under ethylicin treatment (Table 3, Supplementary Table S6B). Interestingly, the important DEPs related to ribosome in GO terms, such as ribosomal protein S9, ribosomal protein L24, ribosomal protein L32, ribosomal protein L27 and ribosomal protein S18, were also enriched in the KEGG pathways of ribosome (Supplementary Table S6B). This was consistent with the analysis of GO terms that ribosome may be the key target inhibited by ethylicin. 

The function of ribosome is major in peptide and protein biosynthesis [15,16]. Thus, the suppression of ribosomal function may cause the inhibition of such biosynthesis. Additionally, the metabolomic analyses show that ethylicin may inhibit amino acid metabolism, leading to the reduction of amino acids in *P. infestans*, and this may also result in the suppression of peptide or protein biosynthesis. Thus, the inhibition of both ribosomal function and amino acid metabolism may result in the suppression of peptide or protein biosynthesis in *P. infestans*. Interestingly, the analyses of child GO terms in proteomics found that the peptide metabolic process was the most significantly altered function among biological processes under treatment by ethylicin (Table 2 and Supplementary Table S5A). All 24 DEPs were down-regulated and none were up-regulated (Table 2 and Supplementary Table S5D), indicating that the peptide metabolic process was inhibited by ethylicin. The peptide metabolic process includes the peptide biosynthetic and catabolic processes. Interestingly, the peptide biosynthetic process was the second most significantly altered function among biological processes (Table 2 and Supplementary Table S5A). However, the peptide catabolic process was not enriched among biological processes, indicating that it was not significantly altered (Table 2 and Supplementary Table S5A). Further analyses found that all 22 DEPs in the peptide biosynthetic process were also enriched in the GO term of the peptide metabolic process, and the 22 DEPs were all down-regulated under ethylicin treatment (Supplementary Table S7). However, no DEPs were enriched in the peptide catabolic process (Supplementary Table S5A). These results suggest that the peptide biosynthetic process is prominently inhibited by ethylicin. Therefore, ethylicin may suppress the peptide or protein biosynthetic process via the inhibition of ribosomal function and amino acid metabolism, thereby contributing to the decreased peptides or proteins in *P. infestans* (T30-4 and 88069 strains). 

**Figure 3:**
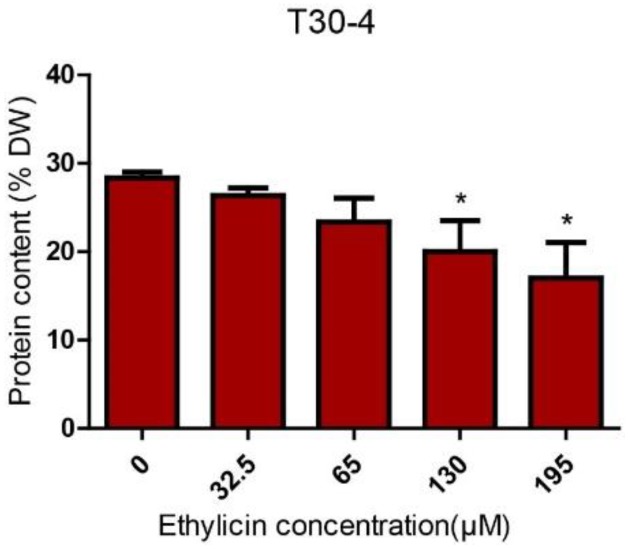
Reduction in protein content after ethylicin treatment (T30-4). Protein content (% DW) = [total protein weight (mg)/mycelia dryweight (mg)] × 100%. Each value represents the mean ± SD of three independent experiments. (* *p* < 0.05, ** *p* < 0.01, *** *p* < 0.001)

In order to confirm the decreased peptides and proteins, we performed a quantitative protein assay to estimate protein content. As we expected, the total protein content in *P. infestans* was significantly reduced after ethylicin treatment in a dose-dependent manner compared with the control group (Figure 3 and Supplementary Figure S2). This result confirms that ethylicin inhibits the synthesis of peptide or protein in *P. infestans*.

#### 3.2.3. Ethylicin May Inhibit Virulence of *P. infestans* by Disrupting Virulence-Related Protein 

**Figure 4:**
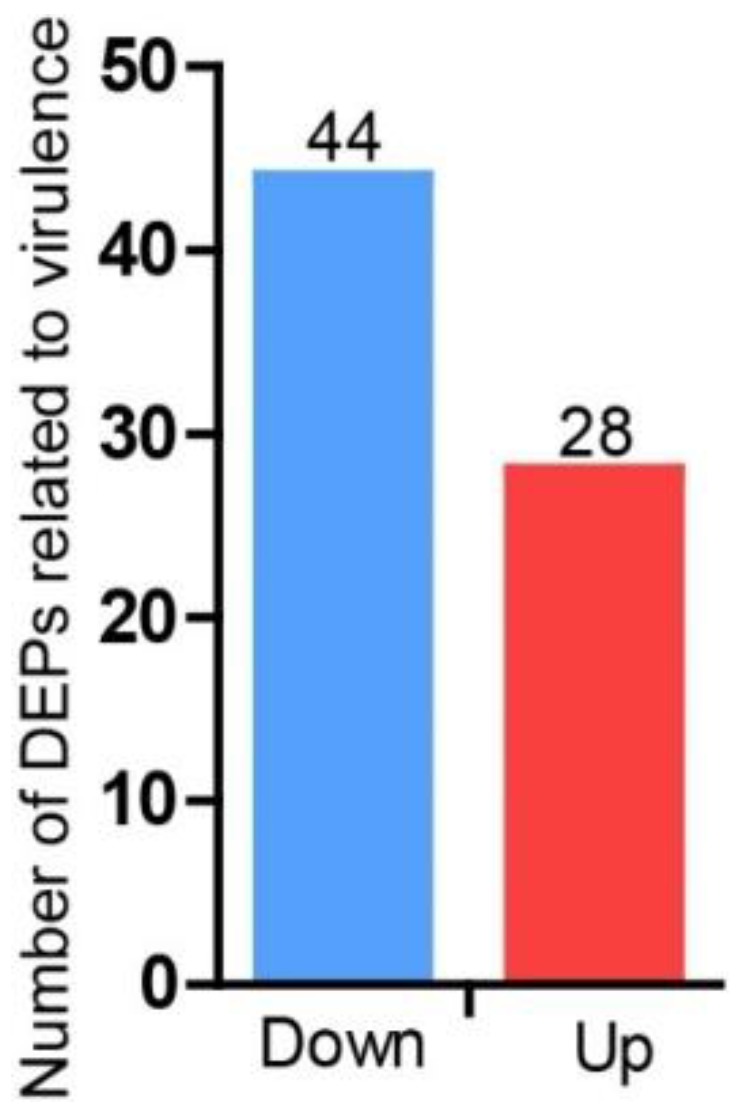
Number of DEPs related to virulence induced by ethylicin. A total of 44 DEPs related to virulence were significantly down-regulated and 28 were up-regulated. Down-regulated DEPs: DEPs with E/DMSO Ratio < 0.833, Up-regulated DEPs: DEPs with E/DMSO Ratio > 1.200 (Supplementary Table S8).

Aside from protein synthesis, virulence-related proteins also contribute to the infection of *P. infestans* on potatoes. To explain the inhibitory effects of ethylicin on virulence, the DEPs related to virulence were analyzed. DEPs related to virulence were searched in the Pathogen–Host Interaction Database (PHI, http://www.phi-base.org/), and a total of 72 DEPs related to virulence were found in the ethylicin-treated samples (Figure 4, Supplementary Table S8). Importantly, 28 DEPs were up-regulated while 44 were down-regulated (Figure 4, Supplementary Table S8), suggesting that virulence-related proteins were obviously inhibited by ethylicin. In particular, important virulence-related proteins such as cytochrome P450, glycoside hydrolase, diaminopimelate decarboxylase and ATP-binding cassette were significantly suppressed by ethylicin (Supplementary Table S8) [17,18,19,20]. Thus, the down-regulation of virulence-related proteins, especially the suppression of key virulence-related proteins, probably contributes to the decreased virulence of *P. infestans* under ethylicin treatment.

## 4. Discussion

In this study, ethylicin was found to exhibit certain inhibitory effects on the mycelial growth, sporulation capacity, spore germination and virulence of *P. infestans*. The integrated analysis of proteomics and metabolomics indicated that ethylicin may suppress both ribosomal function and amino acid metabolism, resulting in an inhibitory effect on peptide or protein biosynthesis in *P. infestans*. These observations reveal the inhibitory effects of ethylicin on *P. infestans* for the first time. 

Metabolomics analysis indicates that ethylicin may inhibit the metabolic process of amino acids and carbohydrates, especially amino acid metabolism, and this may cause the inhibition of peptide or protein biosynthesis due to the decreased substrates (amino acids and energy). This conclusion is consistent with the previous report that ethylicin may interfere with amino acid metabolism in *P. nicotianae*, another oomycetic disease affecting tobacco [21]. However, this previous research only used transcriptome to explore the mechanism [21]. In this research, metabolomics further confirmed the previous conclusion.

In proteomics, the GO terms and KEGG pathway support each other, suggesting that ethylicin may inhibit ribosomal function. Peptide or protein biosynthesis is the main function of ribosome [15,16]. Thus, the inhibition of ribosomal function may cause the suppression of peptide or protein biosynthesis in *P. infestans*. Interestingly, GO term analysis further indicates that peptide biosynthesis is inhibited by ethylicin. This is consistent with the previous report that kasugamycin and oxytetracycline target ribosome to disrupt protein synthesis in pathogens [22,23,24]. In particular, kasugamycin is widely used to control rice blast, which is caused by *Pyricularia oryzae* [25].

**Figure 5:**
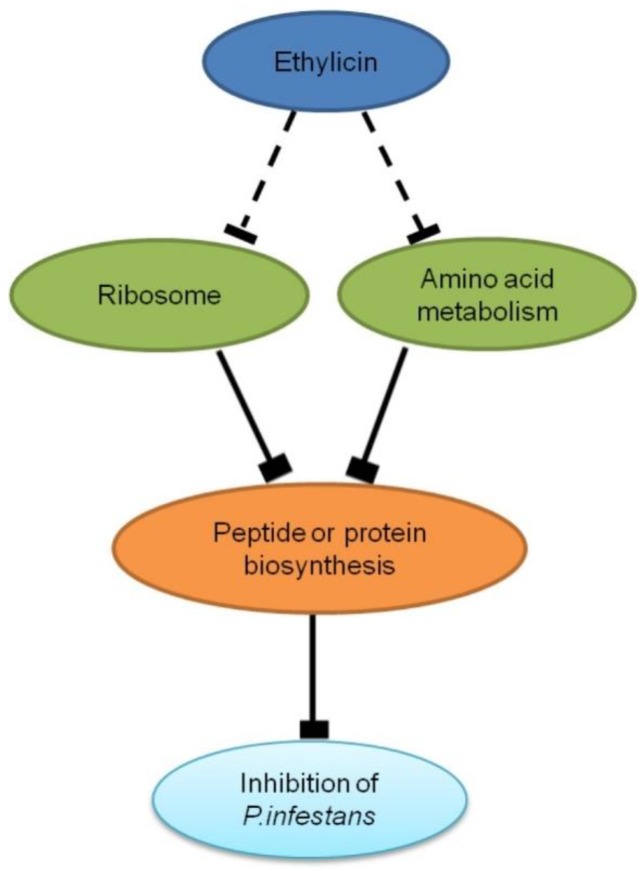
Inhibitory mechanisms of ethylicin on *P. infestans*. Ethylicin may suppress both ribosomal function and amino acid metabolism, leading to the inhibitory effect on peptide or protein biosynthesis in *P. infestans*.

Collectively, ethylicin may inhibit ribosomal function and amino acid metabolism to suppress peptide or protein biosynthesis in *P. infestans*, which was proven by the quantitative protein assay (Figure 5). Interestingly, streptomycin also targets ribosome and amino acid metabolism to disrupt protein biosynthesis in pathogens [26,27,28,29,30]. As the executor of life activities, the reduction of peptide or protein may contribute to the inhibitory effect on a series of biological functions in *P. infestans* [29]. Some fungicides or antibiotics also target peptide or protein biosynthesis to fight against various pathogens, such as chloramphenicol, cycloheximide, anisomycin and blasticidin [31,32]. Thus, peptide or protein biosynthesis is an important target for fungicides or oomyceticides. Previous studies have shown that ethylicin targets cellular molecules with -SH- to inhibit the metabolism of plant pathogens [8]. This study provides new insights into the inhibitory effects of ethylicin on *P. infestans*.

Therefore, this research reveals that ethylicin may inhibit *P. infestans* to some extent, such as mycelial growth, sporulation capacity, spore germination and virulence. Additionally, previous research has shown that ethylicin can promote plant growth, stimulate seed germination and improve quality [8]. Thus, ethylicin has the potential to be developed into biomimetic oomyceticides, which cause less harm to the physiological functions of plants.

## Figures and Tables

**Table 1:** 29 amino acids induced by ethylicin in *P. infestans* (T30-4) in metabolomics.

Name	VIP	Corr.Coeffs	Up (+)/Down (−)	*p*-Value	FC
L-Homoserine	1.5	−0.97	−	7.80 × 10^−8^	0.479
Ratio of Beta-Alanine/L-Aspartic acid	1.5	−0.97	−	1.00 × 10^−7^	0.516
Homocysteine	1.5	−0.97	−	1.30 × 10^−7^	0.516
Beta-Alanine	1.5	−0.97	−	3.40 × 10^−7^	0.54
L-Cysteine	1.5	−0.95	−	2.50 × 10^−6^	0.648
L-Alanine	1.5	−0.94	−	6.20 × 10^−6^	0.817
Ratio of 4-Hydroxyproline/L-Proline	1.5	−0.93	−	1.00 × 10^−5^	0.75
L-Arginine	1.5	−0.92	−	1.70 × 10^−5^	0.636
Methionine sulfoxide	1.4	−0.91	−	5.10 × 10^−5^	0.65
L-Alloisoleucine	1.4	−0.9	−	6.60 × 10^−5^	0.793
Glycine	1.4	−0.89	−	8.60 × 10^−5^	0.761
L-Threonine	1.4	−0.89	−	9.00 × 10^−5^	0.781
D-2-Hydroxyglutaric acid	1.4	−0.89	−	9.90 × 10^−5^	0.633
Ornithine	1.4	−0.87	−	2.10 × 10^−4^	0.759
3-Nitrotyrosine	1.4	−0.87	−	2.10 × 10^−4^	0.588
L-Serine	1.4	−0.86	−	2.90 × 10^−4^	0.788
L-Leucine	1.4	−0.86	−	2.90 × 10^−4^	0.793
4-Hydroxyproline	1.3	−0.83	−	8.80 × 10^−4^	0.727
L-Cystine	1.3	−0.81	−	1.40 × 10^−3^	0.516
L-Valine	1.3	−0.81	−	1.50 × 10^−3^	0.816
L-Methionine	1.3	−0.8	−	1.70 × 10^−3^	0.806
3-Oxoalanine	1.2	−0.74	−	5.70 × 10^−3^	0.478
Ratio of L-Glutamic acid/Pyroglutamic acid	1.1	−0.72	−	7.90 × 10^−3^	0.95
Citrulline	1.1	−0.7	−	1.10 × 10^−2^	0.838
Ratio of Citrulline/Ornithine	1.1	0.68	+	1.50 × 10^−2^	1.104
Ratio of Ornithine/L-Arginine	1.3	0.82	+	1.00 × 10^−3^	1.154
Ratio of Sarcosine/Glycine	1.3	0.82	+	1.10 × 10^−3^	1.248
L-Lysine	1.4	0.87	+	2.30 × 10^−4^	1.226
Ratio of Citrulline/L-Arginine	1.6	0.99	+	2.20 × 10^−9^	1.338

VIP: Variable importance in projection; Corr.Coeffs: Correlation Coefficient; FC: Fold Change; FC > 1: up regulation, FC < 1: down regulation.

**Table 2:** Twenty-six child GO terms in ethylicin-treated samples in proteomics.

GO Terms Level 1	GO Terms Description	Down	Up	*p*-Value
Cellular Component	ribosome	22	0	8.96 × 10^−6^
Cellular Component	cytoplasmic part	34	1	2.77 × 10^−4^
Cellular Component	ribonucleoprotein complex	22	0	3.87 × 10^−4^
Cellular Component	intracellular ribonucleoprotein complex	22	0	3.87 × 10^−4^
Cellular Component	non-membrane-bounded organelle	24	0	4.26 × 10^−3^
Cellular Component	intracellular non-membrane-bounded organelle	24	0	4.26 × 10^−3^
Cellular Component	signal peptidase complex	2	0	8.86 × 10^−3^
Cellular Component	endoplasmic reticulum membrane	4	0	2.05 × 10^−2^
Molecular Function	structural constituent of ribosome	22	1	5.66 × 10^−9^
Molecular Function	structural molecule activity	22	0	1.07 × 10^−7^
Molecular Function	polysaccharide binding	0	3	1.78 × 10^−3^
Molecular Function	pattern binding	0	3	1.78 × 10^−3^
Molecular Function	hydrogen-translocating pyrophosphatase activity	2	0	3.37 × 10^−3^
Molecular Function	cellulose binding	0	2	3.37 × 10^−3^
Molecular Function	hydrolase activity, acting on glycosyl bonds	2	5	1.75 × 10^−2^
Molecular Function	adenylate kinase activity	0	2	1.87 × 10^−2^
Biological Process	peptide metabolic process	24	0	4.39 × 10^−5^
Biological Process	peptide biosynthetic process	22	0	2.25 × 10^−4^
Biological Process	amide biosynthetic process	22	0	7.41 × 10^−4^
Biological Process	cellular macromolecule biosynthetic process	25	2	1.85 × 10^−3^
Biological Process	glucan metabolic process	1	2	1.21 × 10^−2^
Biological Process	cellular polysaccharide metabolic process	1	2	1.21 × 10^−2^
Biological Process	cellular glucan metabolic process	1	2	1.21 × 10^−2^
Biological Process	protein processing	2	0	2.34 × 10^−2^
Biological Process	protein maturation	2	0	2.34 × 10^−2^
Biological Process	cellular carbohydrate biosynthetic process	1	2	4.85 × 10^−2^

GO: Gene Ontology. Down/Up: Number of down-regulated/up-regulated DEPs in corresponding GO terms. Down-regulated DEPs: DEPs with E/DMSO Ratio < 0.833, Up-regulated DEPs: DEPs with E/DMSO Ratio > 1.200. The data on down and up-regulated DEPs is shown in Supplementary Table S4B–D.

**Table 3:** 3 KEGG pathways in ethylicin-treated samples in proteomics.

KEGG Pathway	Down	Up	*p*-Value
Ribosome	24	0	1.42 × 10^−10^
Protein export	4	1	1.34 × 10^−3^
Tyrosine metabolism	2	1	4.81 × 10^−2^

KEGG: Kyoto Encyclopedia of Gene and Genomes. Down/Up: Number of down-regulated/up-regulated DEPs in corresponding KEGG pathways. Down-regulated DEPs: DEPs with E/DMSO Ratio < 0.833, Up-regulated DEPs: DEPs with E/DMSO Ratio > 1.200. The data of down or up-regulated DEPs were shown in Supplementary Table S6B.

## Supplementary Materials

The following are available online at https://www.mdpi.com/2076-0817/9/4/299/s1, Figure S1 Ethylicin significantly inhibits *P. infestans* (88069). (A) Mycelial growth of 88069 treated with different concentrations of ethylicin for 14 days. 65 μM of ethylicin was the IC50 value, while 130 μM completely inhibited mycelial growth. (B) Inhibition rates of ethylicin against mycelial growth for 14 days. (C) Statistics of spore capacity under treatment by different concentrations of ethylicin. (D) Spore germination phenotype of 88069 under treatment by different concentrations of ethylicin. 19.5 μM of ethylicin had a 50% inhibitory effect on spore germination, while 65 μM nearly completely inhibited spore germination. (E) Spore germination rates of 88069 under treatment by different concentrations of ethylicin. (F) Tubers of potato cultivar Favorita were inoculated with spore solutions (88069). Spore solutions were treated by different concentrations of ethylicin. (G) Lesion size of tubers under treatment by different concentrations of ethylicin for 4 days. (H) Disease symptoms of potato plants inoculated with 88069. Potato plants were first sprayed with spores of 88069, then sprayed with different concentrations of ethylicin. Disease symptoms were documented after 10 days. (* *p* < 0.05, ** *p* < 0.01, *** *p* < 0.001). Each value represents the mean ± SD of 3 independent experiments. Figure S2 Reduction in protein content after ethylicin treatment (88069). Each value represents the mean ± SD of 3 independent experiments. Table S1 Disease index and effects of prevention after ethylicin treatment. Table S2 (A) DEMs in ethylicin-treated sample; (B) Insignificant-regulated metabolites Table S3 Number of DEMs in ethylicin-treated sample. Table S4 DEPs in ethylicin-treated sample. Table S5 (A) Child GO terms in 3 kinds of GO term at level 1 in ethylicin-treated sample; Child GO terms in the GO term at level 1 of (B) cellular components, (C) molecular functions and (D) biological processes respectively. Table S6 (A) KEGG pathways in ethylicin-treated sample; (B) DEPs in KEGG pathways in ethylicin-treated sample. Table S7 Detailed information in GO terms of peptide metabolic process and peptide biosynthetic process. Table S8 DEPs related to virulence induced by ethylicin in Pathogen-Host Interaction database. The proteomics datasets were submitted to PRIDE and the ID number—px-submission #411524; The metabonomics datasets were submitted to MetaboLights and the ID number—MTBLS1659.

## Abbreviations

DEMs: differentially expressed metabolites
DEPs: differentially expressed proteins
KEGG: Kyoto Encyclopedia of Genes and Genomes
GO term: Gene ontology
VIP: Variable importance in projection
Corr. Coeffs: Correlation Coefficient. It means variable reliability; if the value is closer to 1 or −1, the reliability is higher. Corr.Coeffs <0: significantly negative correlation; Corr.Coeffs >0: significantly positive correlation.
**NSI:**: Nanospray Flex™
