# Supplementary material for: Ethylicin Prevents Potato Late Blight by Disrupting Protein Biosynthesis of Phytophthora infestans
Source: Pathogens. 2020 Apr 19;9(4):299. doi: 10.3390/pathogens9040299 (PMC7238019; doi:10.3390/pathogens9040299)
Supplement: Supplementary file 1 [file pathogens-09-00299-s001.zip › supplement Figures and Tables/Table S1.docx]

A: T30-4

| Ethylicin concentration | Disease index | Effect of prevention(%) |
| --- | --- | --- |
| CK | 81.54aA | ------- |
| 19.5μM | 53.95bB | 33.83 |
| 130μM | 42.85cC | 47.77 |
| 195μM | 11.05dD | 86.44 |

| Ethylicin concentration | Disease index | Effect of prevention(%) |
| --- | --- | --- |
| CK | 74.15aA | ------- |
| 19.5μM | 53.82bB | 27.42 |
| 65μM | 28.77cC | 61.20 |
| 195μM | 17.24dD | 76.75 |

B: 88069
