# Supplementary figures and images for: Ethylicin Prevents Potato Late Blight by Disrupting Protein Biosynthesis of Phytophthora infestans
Source: Pathogens. 2020 Apr 19;9(4):299. doi: 10.3390/pathogens9040299 (PMC7238019; doi:10.3390/pathogens9040299)

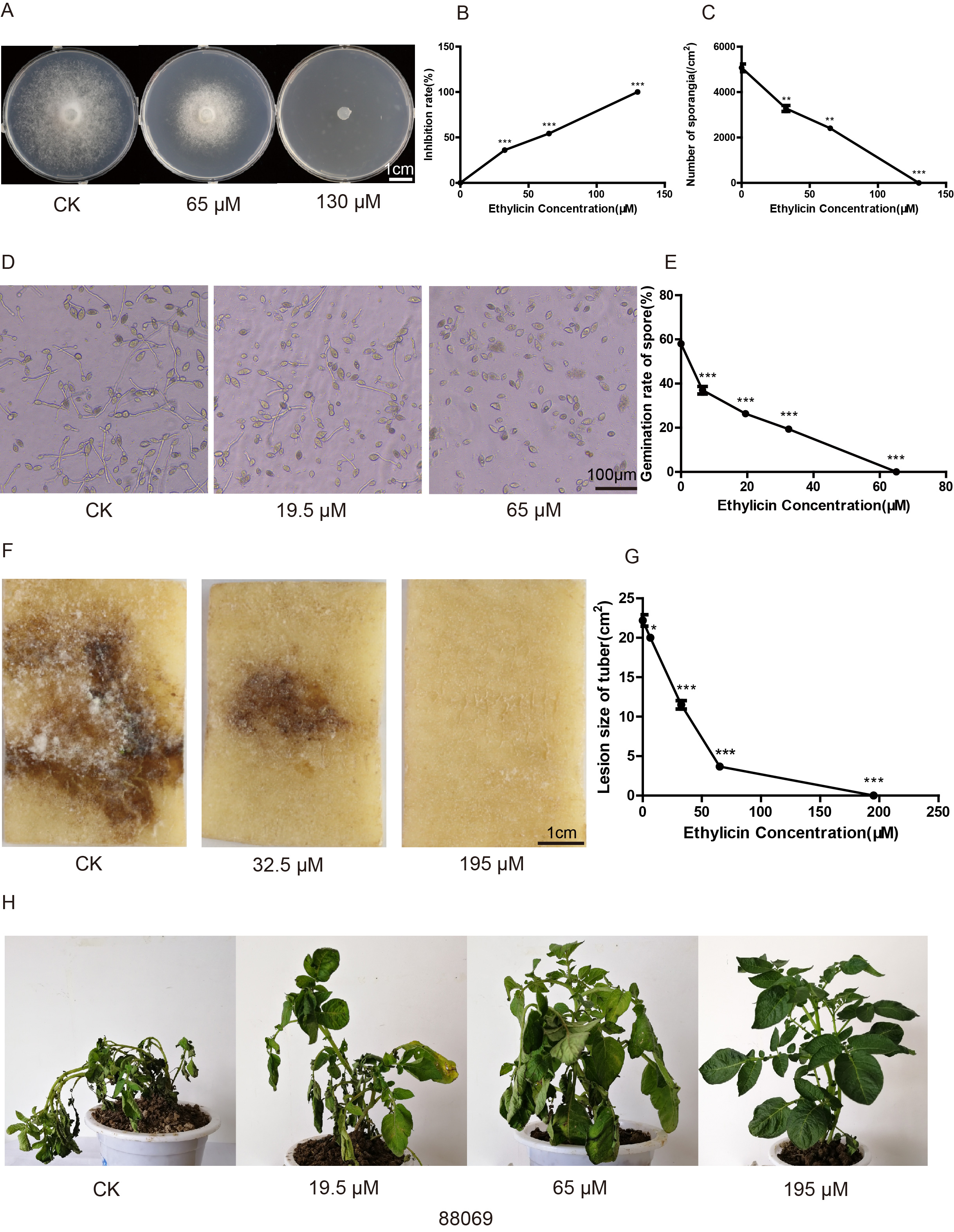

Supplement: Supplementary file 1 [file pathogens-09-00299-s001.zip › supplement Figures and Tables/Fig S1.jpg]

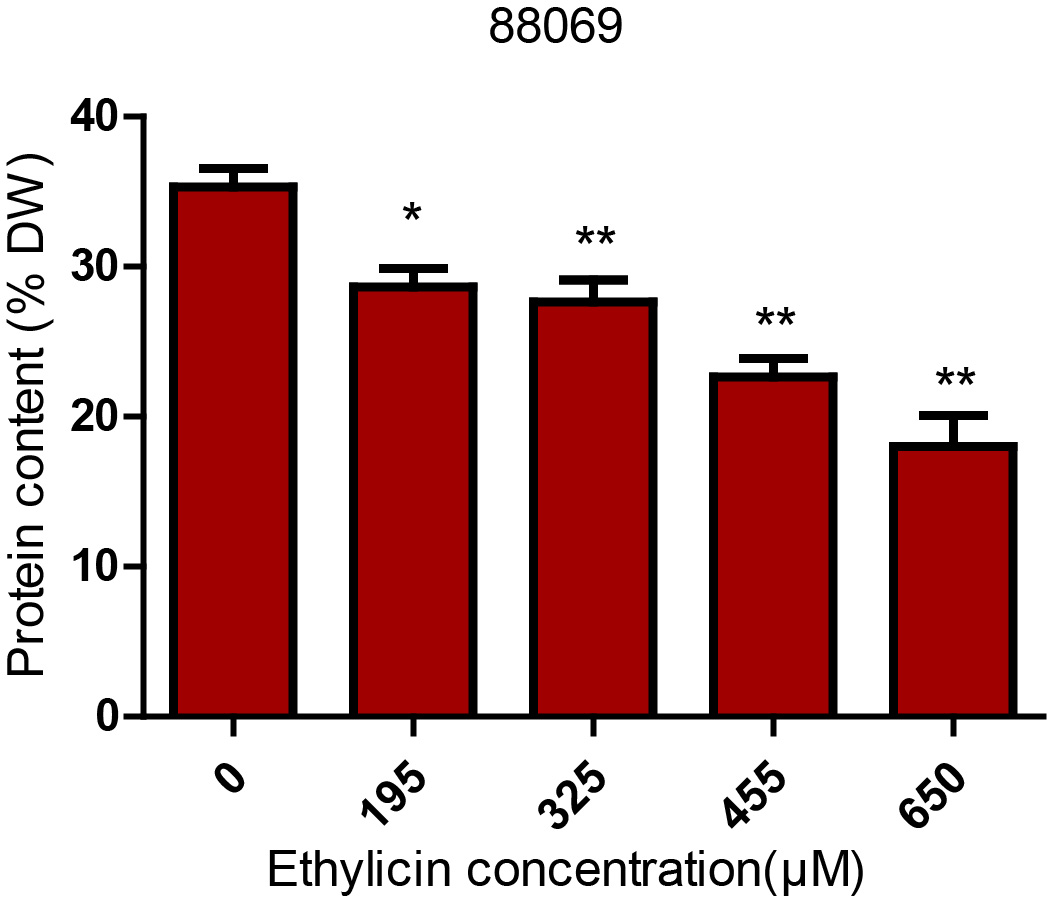

Supplement: Supplementary file 1 [file pathogens-09-00299-s001.zip › supplement Figures and Tables/Fig S2.jpg]
